# Nociceptive Intra-epidermal Electric Stimulation Evokes Steady-State Responses in the Secondary Somatosensory Cortex

**DOI:** 10.1007/s10548-022-00888-y

**Published:** 2022-01-20

**Authors:** Boudewijn van den Berg, Mana Manoochehri, Alfred C. Schouten, Frans C. T. van der Helm, Jan R. Buitenweg

**Affiliations:** 1 Biomedical Signals and Systems, Technical Medical Centre, University of Twente, PO Box 217, 7500 AE Enschede, The Netherlands; 2 Biomechanical Engineering, Faculty of Mechanical, Maritime and Materials Engineering, Delft University of Technology, Delft, The Netherlands; 3 Department of Physical Therapy and Human Movement Sciences, Feinberg School of Medicine, Northwestern University, Chicago, USA; 4 Biomechanical Engineering, Technical Medical Centre, University of Twente, Enschede, The Netherlands

**Keywords:** Intra-epidermal stimulation, Evoked potentials, Steady-state evoked potentials, Nociceptive processing, Source localization, Beamforming

## Abstract

Recent studies have established the presence of nociceptive steady-state evoked potentials (SSEPs), generated in response to thermal or intra-epidermal electric stimuli. This study explores cortical sources and generation mechanisms of nociceptive SSEPs in response to intra-epidermal electric stimuli. Our method was to stimulate healthy volunteers (n = 22, all men) with 100 intra-epidermal pulse sequences. Each sequence had a duration of 8.5 s, and consisted of pulses with a pulse rate between 20 and 200 Hz, which was frequency modulated with a multisine waveform of 3, 7 and 13 Hz (n = 10, 1 excluded) or 3 and 7 Hz (n = 12, 1 excluded). As a result, evoked potentials in response to stimulation onset and contralateral SSEPs at 3 and 7 Hz were observed. The SSEPs at 3 and 7 Hz had an average time delay of 137 ms and 143 ms respectively. The evoked potential in response to stimulation onset had a contralateral minimum (N1) at 115 ms and a central maximum (P2) at 300 ms. Sources for the multisine SSEP at 3 and 7 Hz were found through beamforming near the primary and secondary somatosensory cortex. Sources for the N1 were found near the primary and secondary somatosensory cortex. Sources for the N2-P2 were found near the supplementary motor area. Harmonic and intermodulation frequencies in the SSEP power spectrum remained below a detectable level and no evidence for nonlinearity of nociceptive processing, i.e. processing of peripheral firing rate into cortical evoked potentials, was found.

## Introduction

Nociceptive stimulation leads to an organized response in multiple sensory and cognitive-evaluative brain areas, which is used to study the neurophysiological basis of pain. A temporally well-defined response can be measured when recording the cortical potential on the scalp evoked by a single nociceptive stimulus. This nociceptive evoked potential has a distinct temporal pattern including an early contralateral negative peak (N1), a subsequent central negative peak (N2), and a late central positive peak (P2). This pattern has consistently been reproduced for both laser (Carmon et al. 1978) and intra-epidermal electric (Inui et al. 2002) stimulation of nociceptive afferents. A large body of studies of this temporal pattern found that the N1 is generated by the simultaneous activation of the primary (S1) and secondary (S2) somatosensory cortex (Ploner et al. 1999, 2000; Tarkka and Treede 1993; Valeriani et al. 1996), while the N2-P2 complex appears to be associated with activation of the anterior cingulate cortex (ACC) (Bentley et al. 2002; Garcia-Larrea et al. 2003). Although this pattern is consistently observed in response to nociceptive stimulation, similar activation patterns could be observed in response other stimulation modalities. While the N1 was found to be associated with mostly nociceptive and somatosensory-specific activity, the N2-P2 complex was found to be associated with multimodal activity occurring in response to visual, auditory, somatosensory and nociceptive stimulation (Mouraux and Iannetti 2009). As such, recent studies suggest that the N2-P2 complex is related to the temporal saliency of a stimulus rather than somatosensory or nociceptive specific brain activity (Iannetti and Mouraux 2010).

An alternative approach uses a series of stimuli that are applied at a specific frequency in order to generate a steady-state evoked potential (SSEP). The continuous application of a series of stimuli downregulates the effect of temporal saliency and is thought to result in the entrainment of a network of cortical neurons involved in sensory processing of the stimulus, or the superposition of a series of transient neural responses (Norcia et al. 2015; Picton et al. 2003; Regan 1966). A seminal study by Mouraux et al. used this approach to study pain processing by stimulating participants with blocks of nociceptive laser pulses with the hypothesis that the SSEPs elicited by this rapid thermal stimulation would result in “the activation of a network that is preferentially involved in processing nociceptive input” (Mouraux et al. 2011). Later studies showed that it is also possible to record such a nociceptive SSEP in response to blocks of intra-epidermal electric pulses (Colon et al. 2012) and sinusoidal ultra-slow temperature modulation of the skin (Colon et al. 2017; Mulders et al. 2020).

In a recent study, we showed that it is also possible to evoke nociceptive SSEPs using frequency modulation of intra-epidermal pulses to further downregulate saliency effects (i.e. by decreasing the maximum distance between pulses and avoiding the rapid variations in pulse rate associated with blocks of pulses), and to enable multisine frequency modulation for probing system properties such as system delay, linearity and order (van den Berg et al. 2021). Frequency analysis showed significant peaks at stimulation frequencies (3–7 Hz), but did not find any significant harmonic or intermodulation frequencies that would confirm nonlinearity of nociceptive processing. Using the phase delay of significant nociceptive SSEPs at 3–7 Hz stimulation, we showed that there was an average time delay for both frequencies on the contralateral central midline electrodes (C5, C3, C1 and Cz) of 168 ms which is similar to the average delay of the N1 (160 ms) in previous studies measuring evoked potentials to single intra-epidermal pulses (van den Berg and Buitenweg 2021; van den Berg et al. 2020), suggesting that the nociceptive SSEP might result from the activation of similar neural pathways.

It remains unknown which mechanism and which cortical sources are responsible for the generation of nociceptive SSEPs. In this work, the first objective is to explore if we can identify sources of nociceptive SSEPs and stimulus onset EPs in response to intra-epidermal electric stimulation. In addition, SSEP time delays and EP latencies are compared to explore whether these sources are activated through similar neural pathways. The second objective is to study the (non)linearity of these brain responses based on harmonic and intermodulation frequencies. Comparison of nociceptive SSEP and stimulus onset EP topographies, sources and latencies helps to gain more insight in the functional differences and similarities between transient and steady-state evoked responses in response to nociceptive stimulation.

## Methods

The results presented in this work are based on data from two experiments. The first set of experiments on 10 participants were reported in (van den Berg et al. 2021). Another set of experiments on 12 participants was recorded to extend the original dataset for improved signal-to-noise ratio and source localization.

### Experiment

#### Participants

A total of 22 healthy male volunteers between 18 and 40 years old participated in this study. Only male volunteers were used to prevent potential sex-based differences within the group. All participants provided written informed consent before participation. All experiments were approved by the ethics committee at the Delft University of Technology (approval nr. 1238) and are in accordance with the declaration of Helsinki.

#### Procedure

**Fig. 1:**
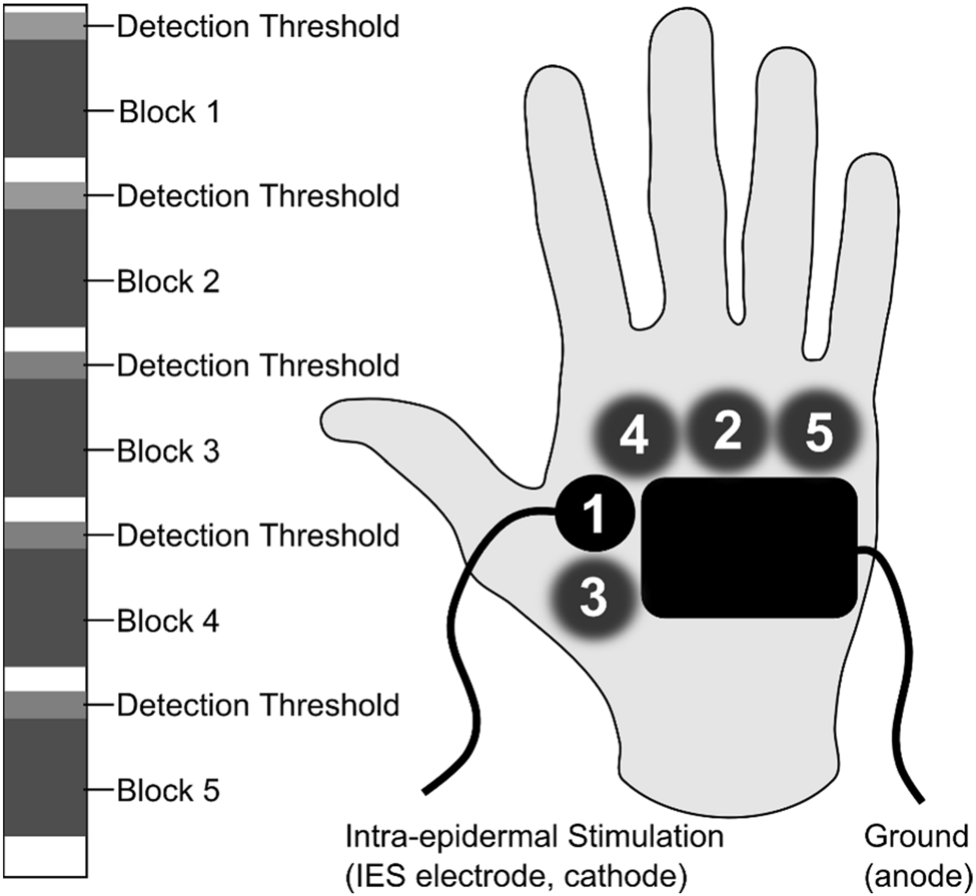
Participants were stimulated in five blocks of 20 frequency modulated pulse sequences. Each block was applied at a different location, indicated by numbers in the figure. In each block, the pulse amplitude was set to twice the detection threshold of a single pulse, measured before the start of each block

Participants were seated upright facing a single neutral image in a dim and silent room. The room was shielded from external electromagnetic interference. Participants were stimulated on the dorsum of the right hand on five different locations (Fig. 1) to reduce potential habituation or sensitization effects induced by repeated stimulation on the same location. On each location, a block of 20 pulse sequences was applied. Each pulse sequence had a duration of 8.5 s. The pulse amplitude during each block was set to twice the detection threshold to a single 0.5 ms pulse. This detection threshold was measured in advance of each block using a staircase paradigm, in which the participant was asked to press and hold a response button, and a single 0.5 ms pulse was applied repeatedly and increased with a stepsize of 0.025 mA (starting from zero) until the participant reported stimulus detection by releasing the response button.

#### Nociceptive Stimulation

**Fig. 2:**
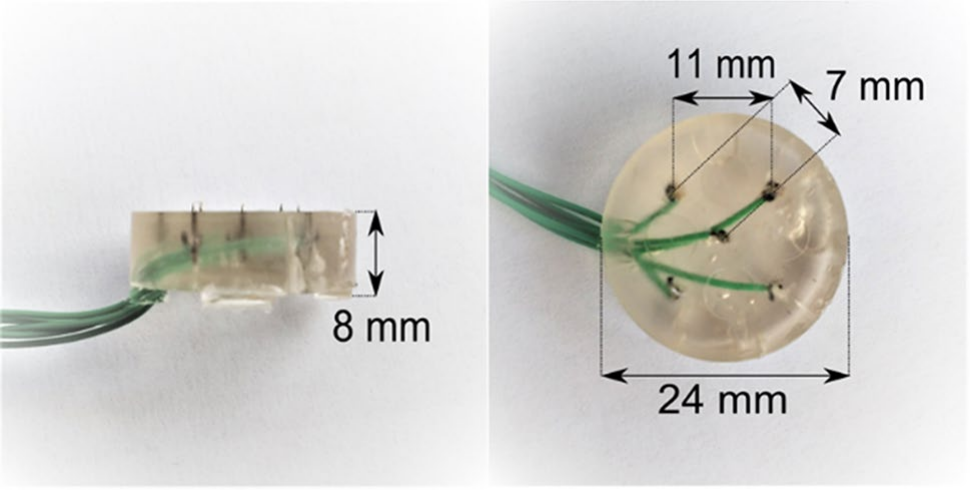
Electrode for intra-epidermal stimulation, consisting of an array of five inter-connected microneedles embedded in a flexible layer of silicone

Intra-epidermal electric stimulation was applied to participants with a current controlled stimulator (AmbuStim, University of Twente, Enschede, the Netherlands) at twice the detection threshold (on average 0.35 ± 0.28 mA). This type of stimulation preferentially activates nociceptive afferents in the epidermis (Mouraux et al. 2010; Poulsen et al. 2020). The stimulation electrode consisted of five microneedles in a layer of flexible silicone (Steenbergen et al. 2012), protruding 0.5 mm from the electrode surface (Fig. 2). Stimulation with this electrode results in a sharp pricking sensation (Steenbergen et al. 2012). The recorded responses using this electrode are similar to other studies using intra-epidermal stimulation (van den Berg and Buitenweg 2021). The electrode was sterilized by autoclave before each measurement.

#### Frequency Modulation

**Fig. 3:**
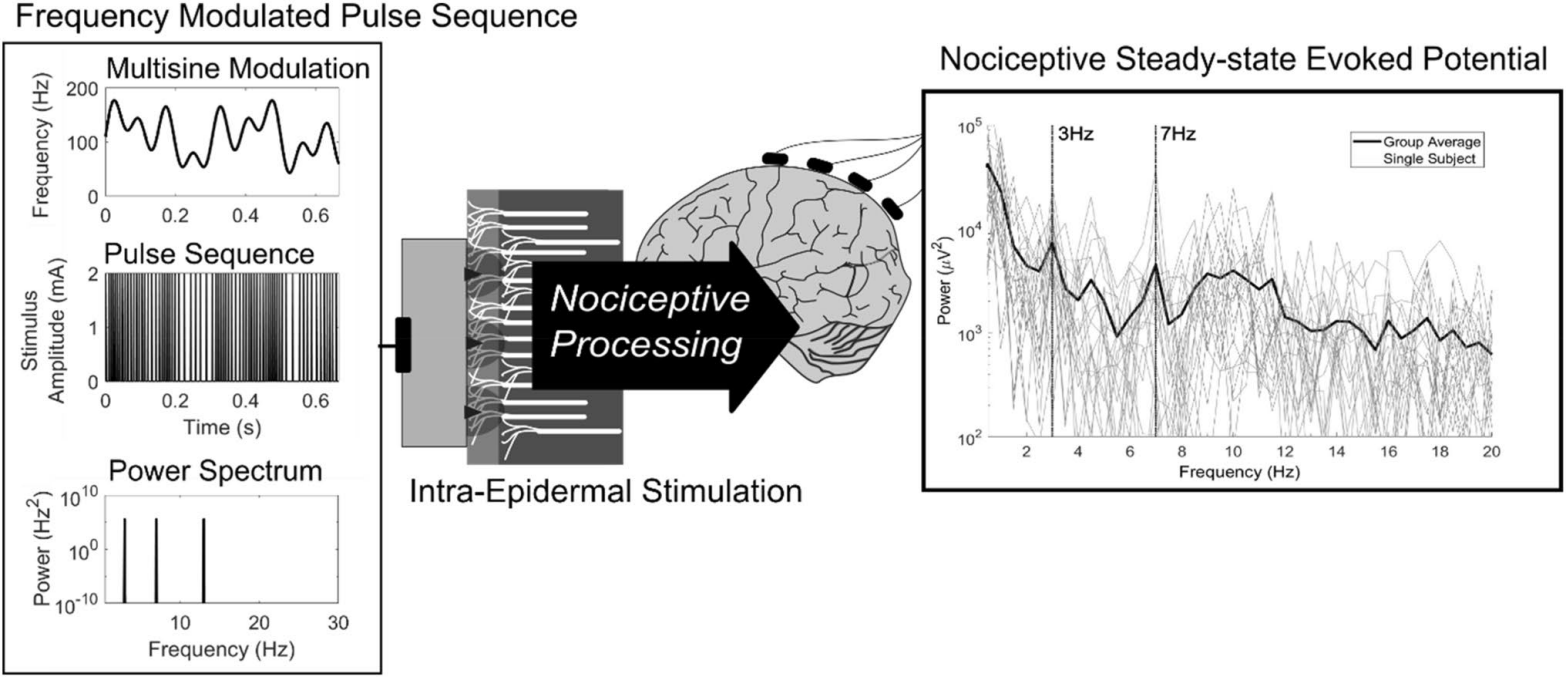
Nociceptive afferents are stimulated with a sequence of intra-epidermal electric pulses. Applying a multisine frequency modulation function (top left) to the frequency of a sequence of electric pulses (middle left) leads to a stimulus with power at the modulation frequencies (bottom left). Stimulation using a multisine frequency modulated pulse sequence leads to a SSEP with peaks at the fundamental stimulation frequencies, harmonics or intermodulation frequencies (right), which can be used to study system (non)linearity and time delay

Stimulation consisted of frequency modulated sequences of square wave cathodic electric pulses, using the same method as was used earlier in (van den Berg et al. 2021). Stimulation was controlled by a microcontroller connected to the trigger input of the stimulator. Each trigger pulse generated by the microcontroller resulted in a single stimulation pulse. Pulse sequences were frequency modulated through modulation of the inter-pulse interval (Fig. 3). Inter-pulse intervals were based on a multisine frequency modulation function, see Eq. (1).

1 $$F_{pulse}\left(t\right)=C_{offset}+A_1\sin\left(2\mathrm\pi F_1t+\phi_1\right)+A_2\sin\left(2\mathrm\pi F_2t+\phi_2\right)+A_3\sin\left(2\mathrm\pi F_3t+\phi_3\right)$$

Applying this multisine frequency modulation function (Fig. 3, top left) to a sequence of electric pulses (Fig. 3, middle left) leads to a stimulus with power at the three modulation frequencies (*F*_1_, *F*_2_, *F*_3_) (Fig. 3, bottom left). Modulation frequencies (*F*_1_, *F*_2_, *F*_3_) were chosen such that measured SSEPs are representative of the behavior of the nociceptive system. In a previous study using square wave intra-epidermal stimuli (Colon et al. 2012) brain responses were measured in a range from 3 to 43 Hz, where lower frequencies resulted in a more consistent response. Furthermore, the frequencies were chosen such that the number of overlapping harmonics and intermodulation frequencies were minimized in order to apply nonlinear system identification techniques to the measured SSEP (Yang et al. 2016), while avoiding frequencies with a large interference by alpha waves. To avoid transient brain activity due to perception of individual pulses within the sequence, the maximum inter-pulse interval was set to 50 ms, i.e. a minimum pulse frequency (*F*_*pulse*_) of 20 Hz. To limit the effects of peripheral nerve repolarization on measured SSEPs, the minimum inter-pulse interval was set to 5 ms, i.e. a maximum pulse frequency (*F*_*pulse*_) of 200 Hz.

For the first 10 participants, modulation frequencies (*F*_1_, *F*_2_, *F*_3_) of 3, 7 and 13 Hz were used, and each modulation amplitude (A_1_, A_2_, A_3_) was set to 30 Hz and phase delays (*ϕ*_1_, *ϕ*_2_, *ϕ*_3_) were set to $0$, $\frac{1}{3}\pi$ and $-\frac{1}{3}\pi$. For the last 12 participants, only the modulation frequencies of 3 and 7 Hz were used to improve SNR by increasing the modulation amplitudes from 30 to 43 Hz, with phase delays set to $-\frac{1}{2}\pi$ and $\frac{1}{2}\pi$.

#### EEG Recording

The scalp EEG was recorded using a TMSi REFA amplifier (TMSi B.V., Oldenzaal, The Netherlands) at a sample rate of 1024 Hz. The signal was recorded at 128 Ag/AgCl electrodes, which were located on the scalp according to the international 10/5 system (Oostenveld and Praamstra 2001). A common average reference was used for recording. In the first ten participants, the ground electrode was located on the right mastoid. In the last 12 participants, the ground electrode was located on the right wrist to minimize possible displacement current artifacts, i.e. artefacts due to the flow of stimulation current to the EEG ground instead of the stimulator ground (McLean et al. 1996). Electrodes were gelled with an impedance below 10 $k{\Omega }$.

### Data Analysis

#### Identification of Stimulation Artifacts

To inspect for stimulation artifacts, a time-locked epoch was extracted around each pulse, with approximately 80,000–100,000 epochs per participant, and high-pass filtered with a cutoff frequency of 60 Hz. The average over all epochs in all sequences and blocks was computed for each participant to obtain the stimulation artifacts. Participants with an average stimulation artifact larger than 100 nV at the Cz channel were excluded.

#### Data Preprocessing

The recorded EEG was pre-processed using EEGlab (Delorme and Makeig 2004). The EEG was high-pass filtered with a cutoff frequency of 1 Hz and low-pass filtered with a cutoff frequency of 40 Hz. To reduce distortion by potential EMG, EOG and movement artifacts, channels in front of the head, on the mastoids and on the lower back of the head were symmetrically removed (M1, M2, FT9, FTT9h, TP7, TPP9h, P9, FT10, FTT10h, TP8, TPP10h, P10, T7, T8, Fp1, Fpz, Fp2, I1, Iz, I2, OI1h, OI2h, AFp3h, AFp4h). In addition, channels with flat or excessive EMG activity were removed from the data (on average two channels per subject). The remaining channels were re-referenced to the common average. Epochs were extracted from − 10.0 to 10.0 s with respect to stimulation onset. Epochs with excessive EMG activity or eye movement artifacts were removed by visual inspection. Any residual contamination by EOG, EMG or movement artifacts was removed using adaptive mixture independent component analysis (Palmer et al. 2008). No contamination by ECG artifacts was found during visual inspection or during independent component analysis.

#### Identification of EPs

For each participant, epochs were averaged across all sequences to identify the evoked potential in response to sequence onset. Channels for EP analysis were selected based on previous publications using intra-epidermal electric stimulation (Liang et al. 2016; Mouraux et al. 2014; van den Berg and Buitenweg 2021). A first negative peak (N1) was defined as the most negative peak at T3-Fz between 80 and 180 ms after stimulus onset. A second negative peak (N2) was defined as the most negative peak at Cz between 100 and 300 ms after stimulus onset. A positive peak (P2) was defined as the most positive peak at Cz between 200 and 500 ms after stimulus onset. To study the average EP waveform across all participants, a grand average EP was computed at T3-Fz and Cz. The grand average EP was tested for significance at N1, N2 and P2 latencies at T3, Cz and T4, and at the T3-Fz derivation. Participants that did not show an N1, i.e. a negative peak between 80 and 180 ms at T3-Fz, were excluded from the grand average and source localization of the N1 waveform. Participants that did not show an N2 or P2, i.e. a negative peak between 100 and 300 ms or a positive peak between 200 and 500 ms at Cz, were excluded from the grand average and source localization of the N2-P2 waveform.

#### Identification of SSEPs

Epochs were limited to 0.5–8.5 s with respect to sequence onset to remove activity evoked by stimulus onset. Epochs were split into four segments of 2 s, giving a total of 400 segments per participant, allowing for additional reduction of spectral noise by averaging, while limiting the frequency resolution to 0.5 Hz. For each participant, the power (${\widehat{|X}\left(f\right)|}^{2}$), phase ($Arg\left(\widehat{X}\left(f\right)\right)$) and noise level ($\frac{\sigma^2\left(f\right)}M$) of time-locked activity across all segments for all central midline electrodes (C3, C1, Cz, C2, C4) were computed. The ${T}_{circ}^{2}$ value (Victor and Mast 1991) was computed across all segments for every channel on every stimulated frequency using Eq. (2).

2 $$T_{circ}^2=\left(M-1\right)\frac{\left|\widehat X\left(f\right)\right|^2}{\sigma^2\left(f\right)}$$

Where $$\widehat X\left(f\right)=\frac1M{\textstyle\sum_{m=1}^M}X_m\left(f\right)$$ and $$\sigma^2\left(f\right)={\textstyle\sum_{m=1}^M}\left(X_m\left(f\right)-\widehat X\left(f\right)\right)^2$$ 

Here, the ${T}_{circ}^{2}$ value is described in terms of the average $\widehat{X}\left(f\right)$ and the variance ${\sigma }^{2}\left(f\right)$ of the Fourier transformed segments ${X}_{m}\left(f\right)$ at frequency $f$ with a total of *M* segments. The group level power spectrum was tested for significance at fundamental stimulation frequencies, harmonics and intermodulation frequencies by testing the ${T}_{circ}^{2}$ against the F-statistic with a significance level of 0.05, as was initially proposed by (Victor and Mast 1991).

#### Source Localization of EPs

Sources of the N1 and the N2-P2 were reconstructed with a linearly constrained minimum variance (LCMV) beamformer (Van Veen et al. 1997) using Fieldtrip (Oostenveld et al. 2011) with a workflow similar to Popov et al. (2018). A forward model was computed using the default volume conduction model provided by Fieldtrip. The average EP waveform was obtained using the full epoch and the covariance matrix was based on the prestimulus activity. Subsequently, LCMV source analysis was performed using ft_sourceanalysis with normalization of the weights to account for the center of the head bias and a regularization parameter of 20%. The individual source reconstructions were averaged over the participants to obtain the grand average brain activity. A mask was used to visualize the top 0.1% of the voxels.

#### Source Localization of SSEPs

Sources of 3 and 7 Hz SSEPs were reconstructed with dynamic imaging of coherent sources (DICS) (Gross et al. 2001), using Fieldtrip (Oostenveld et al. 2011) with a workflow similar to Popov et al. (2018). A forward model was computed using the default volume conduction model provided by Fieldtrip. A dummy signal at the modulation frequency is created for coherence computation. Cross-spectral density matrices are computed for the entire epoch, prestimulus and poststimulus data. A common spatial filter is computed using the cross-spectral density matrix of the entire epoch. Subsequently, source coherence is computed based on prestimulus and poststimulus data using a regularization parameter of 1%. Individual source reconstructions are obtained by computing the coherence difference between prestimulus and poststimulus activity. The individual source reconstructions were averaged to obtain the grand average brain activity. A mask was used to visualize the top 0.1% of voxels.

## Results

A total of 10 participants participated in the first set of experiments and a total of 12 participants participated in the second set of experiments included in this study. Two of the 22 participants were excluded; one due to an excessive stimulation artifact and one due to excessive movement artifacts throughout the experiment.

### Identification of SSEPs

**Fig. 4:**
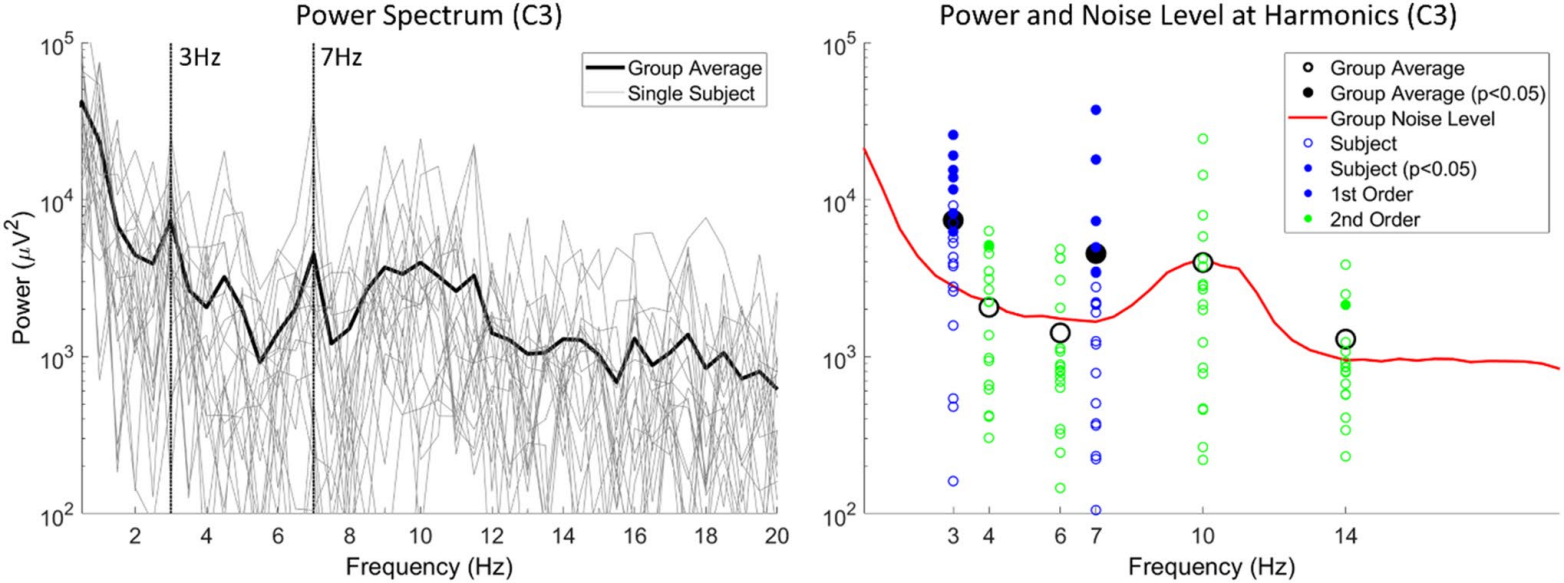
Group and individual steady state evoked potential power spectra at all frequencies (left), and at harmonic and intermodulation frequencies (right) at C3. The power at first order and second order harmonics and intermodulation frequencies in individual participants is shown as blue and green circles, respectively. Group average power at harmonics and intermodulation frequencies is shown in black. Fundamental, harmonic and intermodulation frequencies were tested for significance based on the ${T}_{circ}^{2}$ (see Sect. Identification of SSEPs). Significant frequencies at individual and group level (p < 0.05) are marked with a filled circle. The group average power was significant at 3 and 7 Hz (Color figure online)

**Fig. 5:**
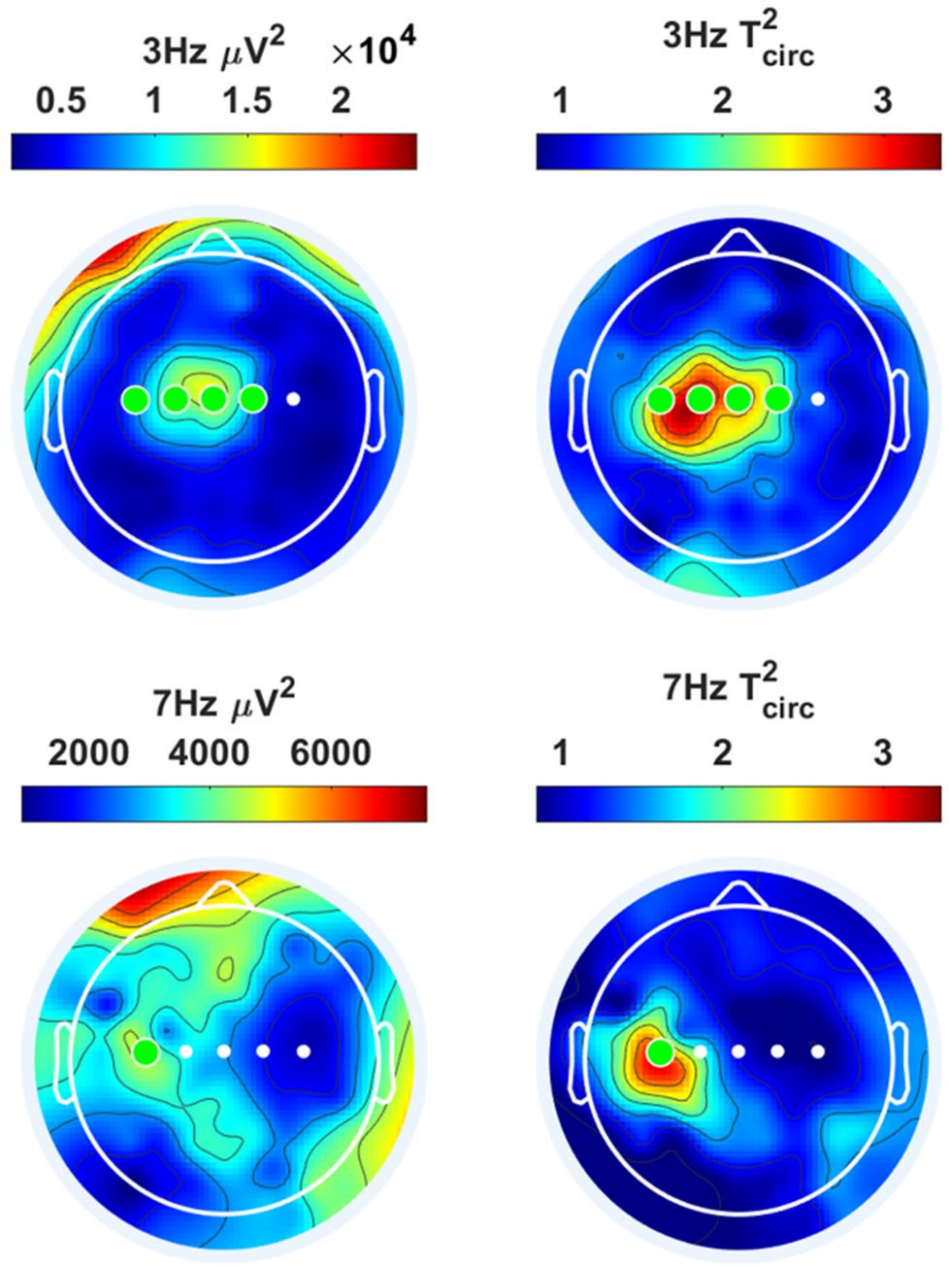
Steady state evoked potential power (left) and ${T}_{circ}^{2}$ (right) topographies at 3 Hz (top) and 7 Hz (bottom). The ${T}_{circ}^{2}$ is an efficient statistic for detecting true SSEP activation out of noise (Norcia et al. 2015). Significance of 3 and 7 Hz at electrodes was tested for significance based on the ${T}_{circ}^{2}$ (see Sect. Identification of SSEPs). Significant electrodes (p < 0.05) are marked by a green dot (Color figure online)

Steady state evoked potential power spectra and topographies are shown in Figs. 4 and 5. Fundamental, harmonic and intermodulation frequencies were tested for significance based on the ${T}_{circ}^{2}$. In addition, significance of 3 and 7 Hz at electrodes was tested for significance based on the ${T}_{circ}^{2}$ (see Sect. Identification of SSEPs). Electrode C3 had significant power (p < 0.05) at 3 and 7 Hz. Other frequencies, including harmonics and intermodulation frequencies, did not have significant power at a group level. For 3 Hz, significant power was found at C2, Cz, C1 and C3. For 7 Hz, significant power was found only at C3. The estimated SSEP delays at C3 was 137.3 ± 22.6 ms at 3 Hz and 143.4 ± 13.7 ms at 7 Hz. For the N1, N2 and P2 there was an average time delay of 142.6 ± 11.9 ms, 192.1 ± 23.0 ms and 459.3 ± 41.8 ms respectively.

### Identification of EPs

**Fig. 6:**
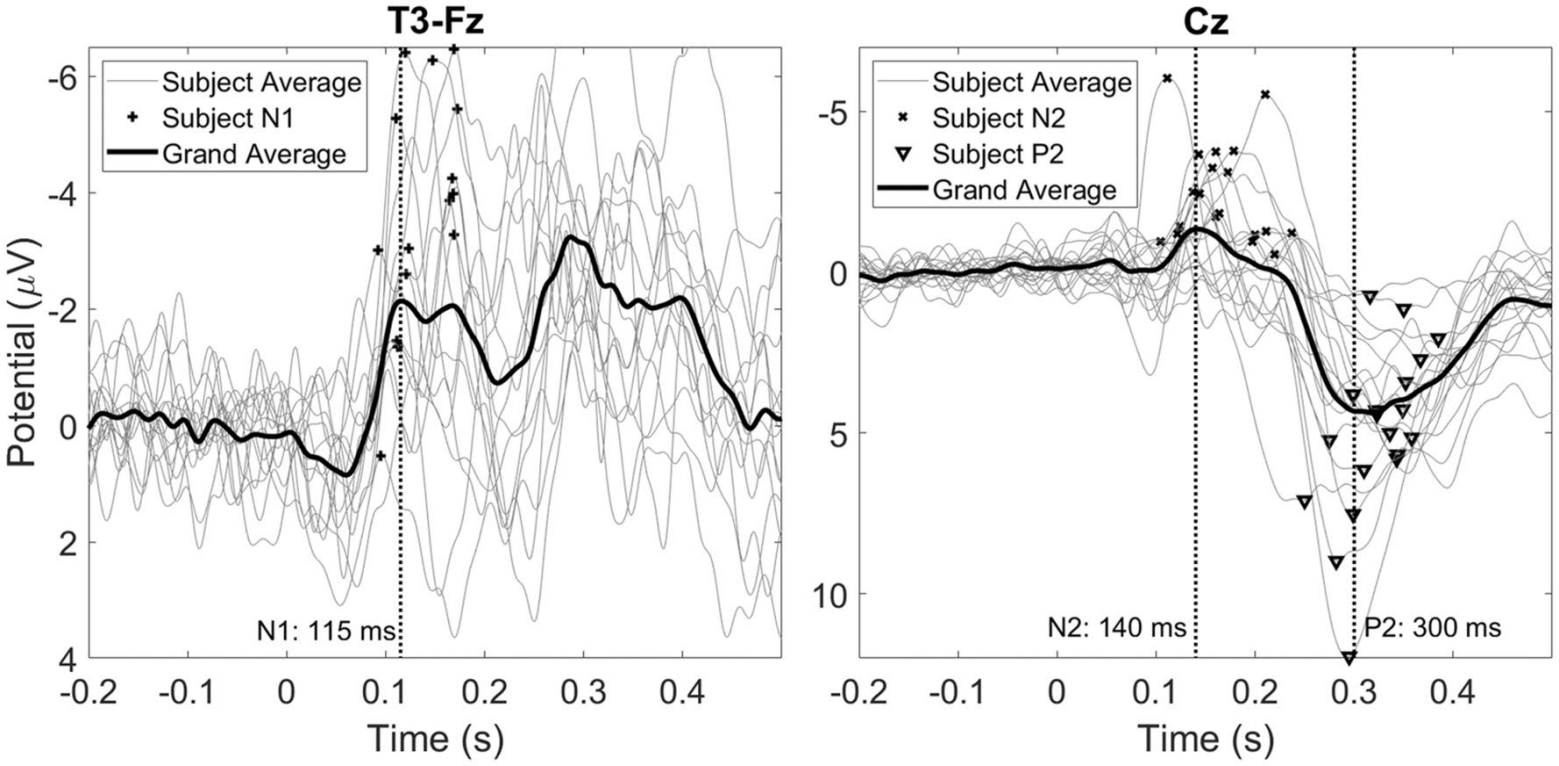
Grand average evoked potential waveforms at T3-Fz and Cz, and the average waveform of each subject. Evoked potential components were defined as the most negative peak on T3-Fz between 80 and 180 ms (N1), the most negative peak at Cz between 100 and 300 ms (N2) and the most positive peak at Cz between 200 and 500 ms (P2). In the grand average, a significant N1 was found at 115 ms (p < 0.01), a significant N2 was found at 140 ms (p < 0.001) and a significant P2 was found at 300 ms (p < 0.001)

**Fig. 7:**
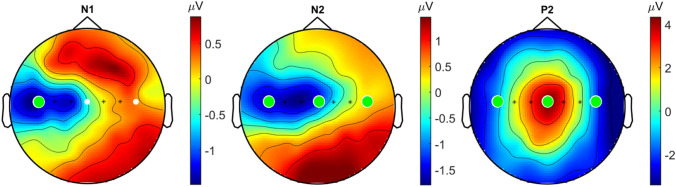
Grand average evoked potential topographies at 115 ms (N1), 140 ms (N2) and 300 ms (P2). The channels T3, Cz and T4 were tested for significance. Significant channels (p < 0.05) are marked by green dots (Color figure online)

**Fig. 8:**
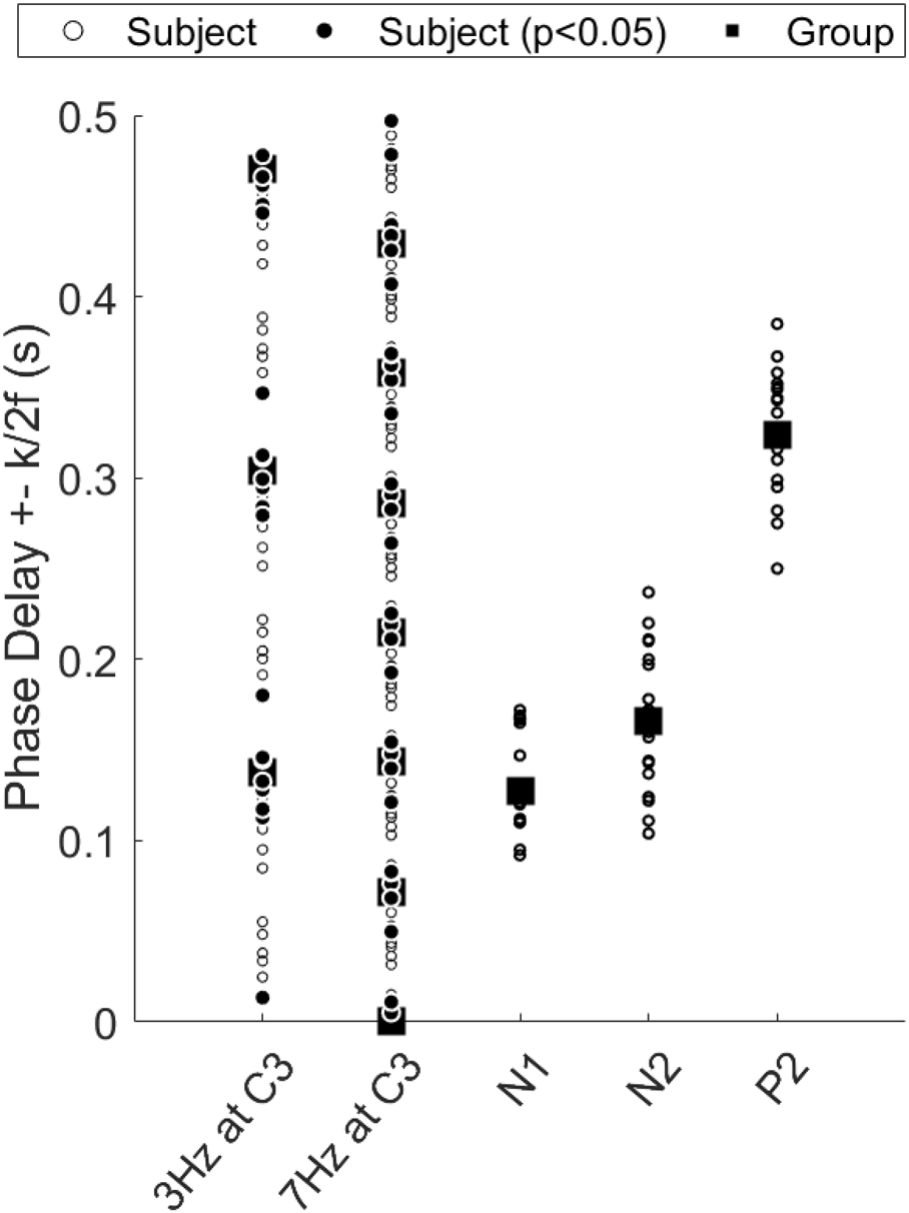
Estimated steady state evoked potential time delay at C3 for 3 and 7 Hz, and the individual and average time delays of N1, N2 and P2 for comparison. There was an average time delay of 137.3 ± 22.6 ms at 3 Hz and an average time delay of 143.4 ± 13.7 ms at 7 Hz. For the N1, N2 and P2 there was an average time delay of 136.1 ± 30.0 ms, 165.8 ± 38.6 ms and 323.9 ± 34.7 ms respectively

Evoked potential waveforms at T3-Fz and Cz are shown in Fig. 6. No N1 was found for a total of five participants. For the remaining 15 participants, a significant (p < 0.01) negative peak between 80 and 180 ms at T3-Fz (N1) was found at 115 ms. The average latency of the N1 was found to be 136.1 ± 30.0 ms. No N2 or P2 was found in one participant. For the remaining 19 participants, a second significant (p < 0.001) negative peak between 100 and 300 ms at Cz (N2) was found at 140 ms. The average latency of the N2 was found to be 165.8 ± 38.6 ms. A significant (p < 0.001) positive peak between 200 and 500 ms at Cz (P2) was found at 300 ms and the average latency of P2 was found to be 323.9 ± 34.7 ms. Grand average topographies of the N1, N2 and P2 in Fig. 7 are showing a contralateral negative potential, a central contralateral potential and a central vertex potential respectively. The N1, N2 and P2 latencies are compared with the SSEP time delay at 3 and 7 Hz in Fig. 8. The P2 latency was significantly later (p < 0.001) than the latencies at 3 and 7 Hz.

### Source Localization of SSEPs

**Fig. 9:**
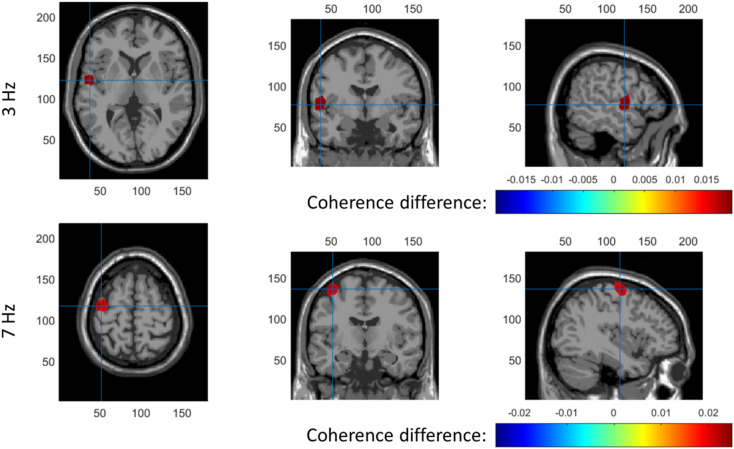
Coronal, transversal and axial slices of reconstructed 3 Hz (top) and 7 Hz (bottom) activity The top 0.1% of voxels is shown in color and the position of the slices is indicated by blue cross hairs (Color figure online)

Coronal, transversal and axial slices of reconstructed 3 and 7 Hz activity are shown in Fig. 9. Position of the slices is indicated by blue crosshairs. The top 0.1% of voxels is shown in color. For 3 Hz, maximum activation was found in the secondary somatosensory cortex and no other sources were found. For 7 Hz, maximum activation was found around the primary motor cortex and no other sources were found.

### Source Localization of EPs

**Fig. 10:**
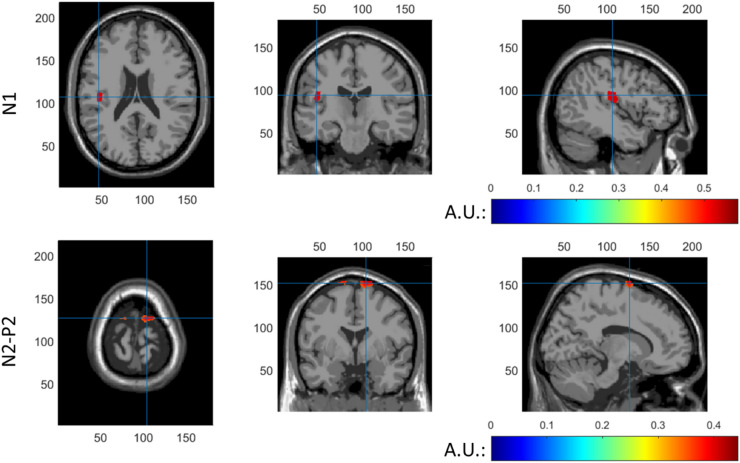
Coronal, transversal and axial slices of reconstructed N1 activity (top) and N2-P2 activity (bottom). The top 0.1% of voxels is shown in color and the position of the slices is indicated by blue cross hairs

Coronal, transversal and axial slices of reconstructed N1 and N2-P2 activity are shown in Fig. 10. Position of the slices is indicated by blue crosshairs. The top 0.1% of voxels is shown in color. For the N1, maximum activation was found in the secondary somatosensory cortex. For the N2-P2, maximum activation was found in the left and right supplementary motor area.

## Discussion

In this study data from over 2000 trials in 20 subjects were combined to study which mechanism and which cortical sources are responsible for the generation of nociceptive SSEPs. The first objective was to explore if we could identify sources of nociceptive SSEPs and stimulus onset EPs in response to intra-epidermal electric stimulation. The second objective was to study the (non)linearity of these brain responses based on harmonic and intermodulation frequencies. We used a multisine waveform of 3 and 7 Hz for frequency modulation of an intra-epidermal electric pulse sequence. We found evoked responses in the time-domain and steady-state evoked responses at 3 and 7 Hz in the frequency domain. We used beamforming to reconstruct sources of nociceptive SSEPs and stimulus onset EPs, and studied system (non)linearity based on the power spectrum.

### Intra-epidermal SSEPs and EPs

Significant group-level SSEPs at 3 and 7 Hz were observed central and contralateral with respect to the side of stimulation, showing that multisine frequency modulation of an intra-epidermal pulse sequence leads steady-state evoked potentials at the stimulated frequencies. Special care was taken to identify and reduce potential stimulation artefacts. Participants with stimulation artifacts larger than 100 nV at Cz were excluded and the remaining stimulation artifact in the first ten participants was centered around the EEG ground (on the right mastoid) and no larger than 50 nV at Cz, while no stimulation artifact was observed in the last 12 participants. As such, the observed SSEPs at 3 and 7 Hz can be attributed to cortical activation in response to intra-epidermal electric stimulation.

The additional value of using multisine modulation instead of modulation at a single frequency, is the possibility to study system nonlinearity and estimate system delay more accurately. As harmonic and intermodulation frequencies remained below a detectable level in the current study, no evidence for nonlinearity of nociceptive processing was found. This is opposite to several SSEP studies in other sensory modalities which clearly demonstrated the presence of nonlinear effects in sensory processing [e.g. in proprioception (Vlaar et al. 2016), in vision (Regan and Regan 1989) and in hearing (Wang et al. 2021)]. In these studies, stimuli were applied to sensory organs that act as a transducer by transforming a form of energy (e.g. work in proprioception, photons in vision, sound waves in hearing) to a neural firing rate. In contrast, in our study the neural firing rate in intra-epidermal electric stimulation is directly modulated through a series of electrical pulses, effectively bypassing nonlinear sensory transduction processes occurring in other sensory modalities. Significant nonlinear effects were absent in this study, potentially as a result of bypassing these sensory transduction processes. Stimulation type and parameters play an important role in system (non)linearity. In the current study, a maximum pulse rate of 200 Hz was used to avoid potential nonlinearities due to the refractory period of nociceptive afferents. The pulse amplitude was kept constant to avoid nonlinear system behavior arising from modulation of the number of recruited nociceptive afferents. Nonlinear behavior could still occur due to nonlinear central neural processing (Roberts and Robinson 2012). The absence of nonlinear behavior in the current experiment implies that central neural processing might be linear around the used stimulation parameters, e.g. due to a linearizing effect of corticothalamic feedback (Song et al. 2021). However, absence of any type of nonlinearity remains difficult to prove, and further research is needed to explore the dynamics of central nociceptive processing.

Earlier SSEP studies hypothesized that SSEPs either originate from the entrainment of neural oscillators or the linear superposition of transient responses (Norcia et al. 2015). There is compelling evidence that, in contrast with the neural entrainment hypothesis, auditory (Bohórquez and Ozdamar 2008; Bohorquez et al. 2007; Galambos et al. 1981; Santarelli et al. 1995) and visual (Capilla et al. 2011) SSEPs are generated by the linear superposition of transient responses to the stimuli. As such, it is not unlikely that nociceptive SSEPs could also be explained by the linear superposition of N1 responses. The current results cannot be used to confirm one of both hypotheses [e.g. as is done in (Capilla et al. 2011)], due to absence of jittered control sequences. However, observation of an overlap in location and delay of SSEP and transient responses could warrant further investigation of the linear superposition hypothesis. The transient response evoked by intra-epidermal electric stimulation was analyzed by averaging trials around stimulus onset. A significant N1 wave and N2-P2 wave were found. Topographies and timing of both waves match earlier articles involving intra-epidermal electric stimulation (Mouraux et al. 2014; van den Berg and Buitenweg 2021). Whereas the delay of the P2 was significantly later (p < 0.001) than the delay of 3 and 7 Hz SSEPs, the delays of the N1 (136.1 ± 30.0 ms) and the N2 (165.8 ± 38.6 ms) were close to the delay observed at 3 (137.3 ± 22.6 ms) and 7 Hz (143.4 ± 13.7 ms), suggesting that similar neural processing pathways are involved in the generation of the nociceptive intra-epidermal SSEPs and EPs.

### Source Localization of Intra-epidermal SSEPs

For the first time, this study localized cortical sources of SSEPs in response to intra-epidermal electric stimulation. A major obstacle for accurate source localization was the low signal-to-noise ratio of the signal caused by both the low stimulus amplitude required to preferentially stimulate nociceptive afferents and the division of bandwidth over multiple stimulation frequencies to probe system (non)linearity. For a more elaborate discussion of parameters involved in the signal-to-noise ratio, please refer to (van den Berg et al. 2021). As a result, individual source estimates (not reported) suffered from noise and were not accurately depicting a single source location. However, by averaging individual source estimates, group-level sources converged to areas at the secondary somatosensory cortex (3 Hz) and the primary motor cortex (7 Hz).

The source of the SSEP at 7 Hz was located around the primary motor cortex (M1). Earlier nociceptive and pain studies did occasionally report M1 activation, suggesting that this activation could be related to suppression of movement or pain-evoked movements (Apkarian et al. 2005). Both explanations seem unlikely in this study, as participants generally experience intra-epidermal SSEPs as non-painful (van den Berg et al. 2021). Earlier nociceptive and pain studies using EEG and MEG did consistently report sources in S1 and S2. As such the source observed in M1 could also be explained by a biased estimate of S1 location. A potential bias in source locations in this study is caused by the use of a standard leadfield and MRI model.

The source of the SSEP at 3 Hz was located at the secondary somatosensory cortex. Activation of S2 is frequently observed in both EEG, MEG and fMRI studies, in response to both phasic and tonic stimulation. Both S1 and S2 are thought to be involved in parallel in upstream nociceptive sensory processing before the signal is passed on to the ACC for the cognitive-evaluative stages of pain processing (Apkarian et al. 2005; Ploner et al. 1999). The identification of the S2 as a generator of nociceptive intra-epidermal SSEPs suggests that these reflect sensory-discriminative rather than cognitive-evaluative aspects of nociceptive processing.

A comparison was made between the aforementioned SSEP sources and sources of the stimulus onset EP. Source localization using a LCMV beamformer identified the S2 as a primary source of the N1. Several previous MEG studies reported parallel activation of both S1 and S2 in response to nociceptive stimulation, e.g. (Ninomiya et al. 2001; Ploner et al. 1999, 2000). Although some EEG studies also observe activation of both sources, as in (Valentini et al. 2012), many other EEG studies observe sources in only the S2 (Apkarian et al. 2005). One possible reason for not observing both sources is the high correlation between both sources, which does not only make it more difficult to observe distinct sources using beamforming (Belardinelli et al. 2012), but also violates the independence assumption of independent component analysis. In the current study, we did not observe N1 sources in S1, but the reconstruction of sources in the S1 could have been impeded by the presence of highly correlated sources in the S2. Source localization using a LCMV beamformer identified bilateral activity in the supplementary motor area as a potential source of the N2-P2 waveform. Several earlier studies reported activation of the supplementary motor area in response to painful stimulation (Christmann et al. 2007; Peyron et al. 2000), which might be attributed to preparation or inhibition of motor reactions. While earlier studies also identified the ACC as a primary generator of the N2-P2 waveform (Bentley et al. 2002; Garcia-Larrea et al. 2003), the current study could not find evidence for contribution of the ACC to the N2-P2 waveform. One potential reason is that ACC activation by painful stimuli could be confounded by supplementary motor activity (Apkarian et al. 2005), in which case activity from the superficial supplementary motor area might contribute more to the observed EEG waveform than deep sources like the ACC. As such, source analysis suggests that sources of the N1 waveform and of the SSEP at 3 Hz are located in the same region, while the N2-P2 waveform associated with stimulus onset appears to originate from different regions of the brain.

### Limitations

This was one of the first studies to reconstruct sources of nociceptive SSEPs. Despite the interesting possibilities nociceptive SSEPs might offer to study nociceptive processing, few studies have been performed on this topic as it remains challenging to reliably generate nociceptive SSEPs. In the case of intra-epidermal electric stimulation, stimulation parameters are adjusted for preferential activation of nociceptive afferents. In the first studies using intra-epidermal stimulation, preferential activation was achieved by a concentric needle electrode (Inui and Kakigi 2012; Inui et al. 2002). In the current study, we used a silicone needle electrode together with a separate planar ground electrode to stimulate nociceptive afferents. Stimulation using this configuration was shown to result in a sharp pricking sensation (Steenbergen et al. 2012) and to result in reaction times and evoked response latencies similar to earlier studies using laser or intra-epidermal stimulation (van den Berg and Buitenweg 2021), indicating preferential activation of nociceptive afferents. However, in order to remain preferential, both configurations require a limitation of stimulus intensity to twice the detection threshold (Mouraux et al. 2010; Poulsen et al. 2020) to reduce concurrent activation of tactile afferents, which leads to a reduction in signal-to-noise ratio of the SSEP and challenges conventional signal analysis and source localization approaches. Therefore, the current techniques demand for ideal experimental circumstances to reliably observe nociceptive brain responses, including optimized setup and stimulation parameters, as well as a homogeneous study population.

The aim of this study was to explore whether it is possible to reconstruct sources of the nociceptive SSEP under these ideal circumstances. These ideal circumstances include a homogeneous study population, as it is well-known that age or sex can influence pain outcomes (Bartley and Fillingim 2013) and might potentially affect evoked brain potentials (Monciunskaite et al. 2019), and as a consequence reduce significance on a group level in a mixed population. For this reason, the current study was done with a homogeneous population of young healthy males. It is therefore important to note that the generalizability of these results might be limited, and that these results should be reproduced in populations of different age, sex and ethnicity to provide more inclusive evidence of the effects observed in this study.

## Conclusions

This study aimed to explore sources of nociceptive SSEPs and stimulus onset EPs following intra-epidermal electric stimulation, and to study the (non)linearity of these brain responses. We found that potential sources of nociceptive SSEPs can be studied through beamforming, and that these sources are located at or near the somatosensory cortices. We observed EPs in response to stimulation onset that were similar to the EPs evoked by a single electric or laser pulse, and the sources of these EPs were located at the secondary somatosensory cortex and the supplementary motor area. Multisine frequency modulation of the applied intra-epidermal pulse sequences leads to cortical activation at the stimulated frequencies. As harmonic and intermodulation frequencies remained below a detectable level in the current study, no evidence for nonlinearity of nociceptive processing was found.

## Data Availability

All data of the reported experiments are available without restrictions on request from the corresponding author. The provision of data complies with local ethics review and the mandates of the study funder (NWO).

## Code Availability

All code of the reported analyses is available without restrictions on request from the corresponding author. The provision of code complies with local ethics review and the mandates of the study funder (NWO).
